# Plaid masking explained with input-dependent dendritic nonlinearities

**DOI:** 10.1038/s41598-024-75471-5

**Published:** 2024-10-22

**Authors:** Marcelo Bertalmío, Alexia Durán Vizcaíno, Jesús Malo, Felix A. Wichmann

**Affiliations:** 1 Spanish National Research Council (CSIC), Madrid, Spain; 2 Universitat de València, València, Spain; 3 University of Tübingen, Tübingen, Germany

**Keywords:** Psychology, Computational neuroscience, Computational neuroscience, Neuroscience, Perception

## Abstract

A serious obstacle for understanding early spatial vision comes from the failure of the so-called standard model (SM) to predict the perception of plaid masking. But the SM originated from a major oversimplification of single neuron computations, ignoring fundamental properties of dendrites. Here we show that a spatial vision model including computations mimicking the input-dependent nature of dendritic nonlinearities, i.e. including nonlinear neural summation, has the potential to explain plaid masking data.

## Introduction

Spatial vision as a field is concerned with the fundamental question of how patterns of light on the retina are encoded, and how the encoded activity is then transformed along the first stages of visual processing—on an algorithmic level in Marr’s terminology^1^. Inspired by the seminal work of Campbell and Robson^2^, classic studies^3–7^ and many subsequent efforts (see Ref.^8^) have led to what has been called a *standard model* (SM) of spatial vision: A linear multi-scale decomposition into spatial frequency and orientation specific channels followed by nonlinear divisive normalisation within and across channels. The SM has shown to be remarkably successful, and its latest image-computable incarnations are able to predict, simultaneously with the same set of parameters, a variety of classical sinusoidal contrast detection and discrimination experiments as well as the detectability of grating patches against natural images^9–11^.

However, it has also become apparent that the SM is unable to account for a number of psychophysical studies using simple grating stimuli which it *ought* to be able to account for^12–15^. Perhaps the most striking failure of the SM is its inability to explain the plaid masking data reported by Derrington and Henning in 1989^16^ —highlighted in the literature as a significant obstacle to our full understanding of early spatial vision^17^. In the first experiment in^16^ the authors measure the detectability of a vertically oriented sinusoidal grating signal presented in three different situations: with no masker (against a uniform field); with a single masker, a grating oriented $45^\circ$ from the vertical; and with two maskers—a “plaid masker”—with two gratings oriented $\pm 45^\circ$ from the vertical. In the experiment the masker contrast is kept constant and the signal contrast is changed so as to measure the detectability threshold contrast in a temporal 2AFC paradigm. The authors define the “threshold elevation factor” (TEV) as the ratio of the threshold with masking to the unmasked threshold. The results of the experiment show that the TEV for a single masker is around 2.2—perfectly consistent with the SM—while for two maskers it is much higher, around 9: Strongly super-additive masking not explicable in the SM. As Derrington and Henning elegantly put it, these results are *“difficult to reconcile with the notion of signal detection through mechanisms tuned to narrow ranges of orientation”*.

The second experiment measures the effect of masking when the spatial frequency of the signal varies and the spatial frequency of the plaid maskers (with components oriented $\pm 22.5^\circ , \pm 45^\circ$ and $\pm 67.5^\circ$ either side of vertical) is kept constant. According to the SM, the orientation tuning of the visual channels should make the $\pm 22.5^\circ$ maskers produce masking, the $\pm 45^\circ$ maskers reduced masking, and the $\pm 67.5^\circ$ maskers virtually none. But the results in^16^ show this clearly not to be the case, as there is almost as much masking with $\pm 67.5^\circ$ maskers as there is with $\pm 22.5^\circ$ maskers. The fact that the maximum masking effect is very close to the spatial frequency of the components of the masker rules out that the masking could be produced by a pre-striate static nonlinearity, as explained in detail by the authors, who conclude their paper with a sobering note about *“the futility of attempting to explain the relatively simple visual discrimination results we present with a model based on linear mechanisms tuned for spatial frequency and orientation”*: that is, the SM.

The severe problem of the plaid masking data for the SM model, and, consequently, for our concept of spatial vision as a linear-nonlinear (LN) cascade, remains fully open today, as a modern state-of-the-art early vision model by Schütt and Wichmann from 2017^9^ still cannot predict plaid masking data. Thus, plaid masking remains a major obstacle to our understanding of early spatial vision, 35 years after its discovery.

We conjecture that the main reason behind the abovementioned failures is that the (psychophysical) SM originated from a simplified model of biological neurons: the standard neuron model ignores important properties that we now—decades after its introduction—know are fundamental for predicting neural responses, namely the nonlinear, dynamic and input-dependent nature of dendritic computations^18^. Specifically, LN cascade models either assume that dendrites behave linearly (i.e. the “point-neuron” model^19–21^), or they have a multi-stage LN structure of nonlinear subunits that allows to consider dendritic nonlinearities^22–26^ but disregards a key property of dendritic processing which is that dendrites receive backpropagating action potentials (bAPs) initiated from the soma^18,27^. The main obstacle for taking into account bAPs in these models is that bAPs allow the cell output to interact with the input, thus turning the activation of the neuron into a complicated function that cannot be represented in a practical manner under an LN formulation^28^. As a result the SM for neural activity has been shown, like the psychophysical SM, to be more limited in its predictivity than envisaged^29–34^. In particular, given that dendritic properties are key to facilitate neural adaptation to the input (leading to more efficient information representation, more robust information processing and metabolic savings), thus the SM for single neurons, by assuming very simplistic dendritic behaviour, is ill-suited to explain adaptation phenomena. In practice this is typically compensated by changing the model’s parameter values depending on the input to be able to reproduce data observed in real neurons^35–38^.

As an alternative to the LN formalism for single-neuron nonlinear summation, Bertalmío et al.^39^ introduced the Intrinsically Nonlinear Receptive Field (INRF) model, which considers both dendritic nonlinearities and bAPs. This model and a recent extension^40^ have been shown to explain a number of properties of cortical cells that challenge standard approaches. At the same time, the INRF formulation as a *psychophysical model* with a fixed set of parameters can explain several challenging visual perception phenomena for which the SM parameters must change with the input^39^, such as the “crispening” effect^41^, the irradiation illusion^42^ or White’s illusion under noise^43^; and a very recent spatio-temporal version of INRF shows promising results in motion detection^44^. The potential of INRF to contribute towards an accurate model of perception has been further reinforced by the excellent results of image and video quality metrics based on it^45^: here the INRF model acts as a “perceptual transform” that maps the original image to a representation that emulates how it is subjectively perceived.

From a mathematical point of view the INRF model is more flexible than the SM at approximating nonlinear functions, as it uses not a static but an input-dependent nonlinearity. Given that the failure of the SM to account for plaid masking data is essentially a failure to be not “nonlinear enough”, we tested whether the INRF model could explain human plaid masking.

## Methods

### Modeling nonlinear dendrites

 In our experiments we have used the Intrinsically Nonlinear Receptive Field (INRF) model of single-neuron nonlinear summation introduced in Ref.^39^. The INRF equation for the response *O* of the *i*-th neuron (located at spatial location *i*) to a static 2D input signal *I* is:

1 $$\begin{aligned} O_i= \sum _j m_{ij}I_j -\lambda \sum _j w_{ij} f ( I_j - \sum _k g_{ik}I_k ), \end{aligned}$$

where *m*, *w*, *g* are 2D kernels, *j*, *k* are the indices of neighboring locations, $\lambda$ is a scalar, and $f:\mathbb {R}\rightarrow \mathbb {R}$ is a nonlinear function.

We have decided to instantiate the INRF model with elements that are consistent with retinal physiology: *m*, *w*, *g* are chosen to be 2D Gaussians with respective standard deviations $\sigma _m,\sigma _w,\sigma _g$, and we set *f* to be a sigmoid function of the form $f(z)=\frac{2}{1 + e^{-pz}} -1$, wth *p* denoting the slope at zero. All in all we have 5 parameters to set: $\sigma _m,\sigma _w,\sigma _g,\lambda ,p$.

In all experiments the procedure is the same: given two images, one consisting of a sinusoidal grating signal plus a mask (oblique or plaid), and the other consisting of the mask alone, we apply our chosen instance of the INRF model to both images and then compute the RMSE between the results: this value will be our proxy for the perceived visual difference between the two test images. The detectability “threshold” contrast for the grating signal over uniform background is estimated to be 0.0025 based on the data from Ref.^16^ for the 3 cpd case.

**Fig. 1:**
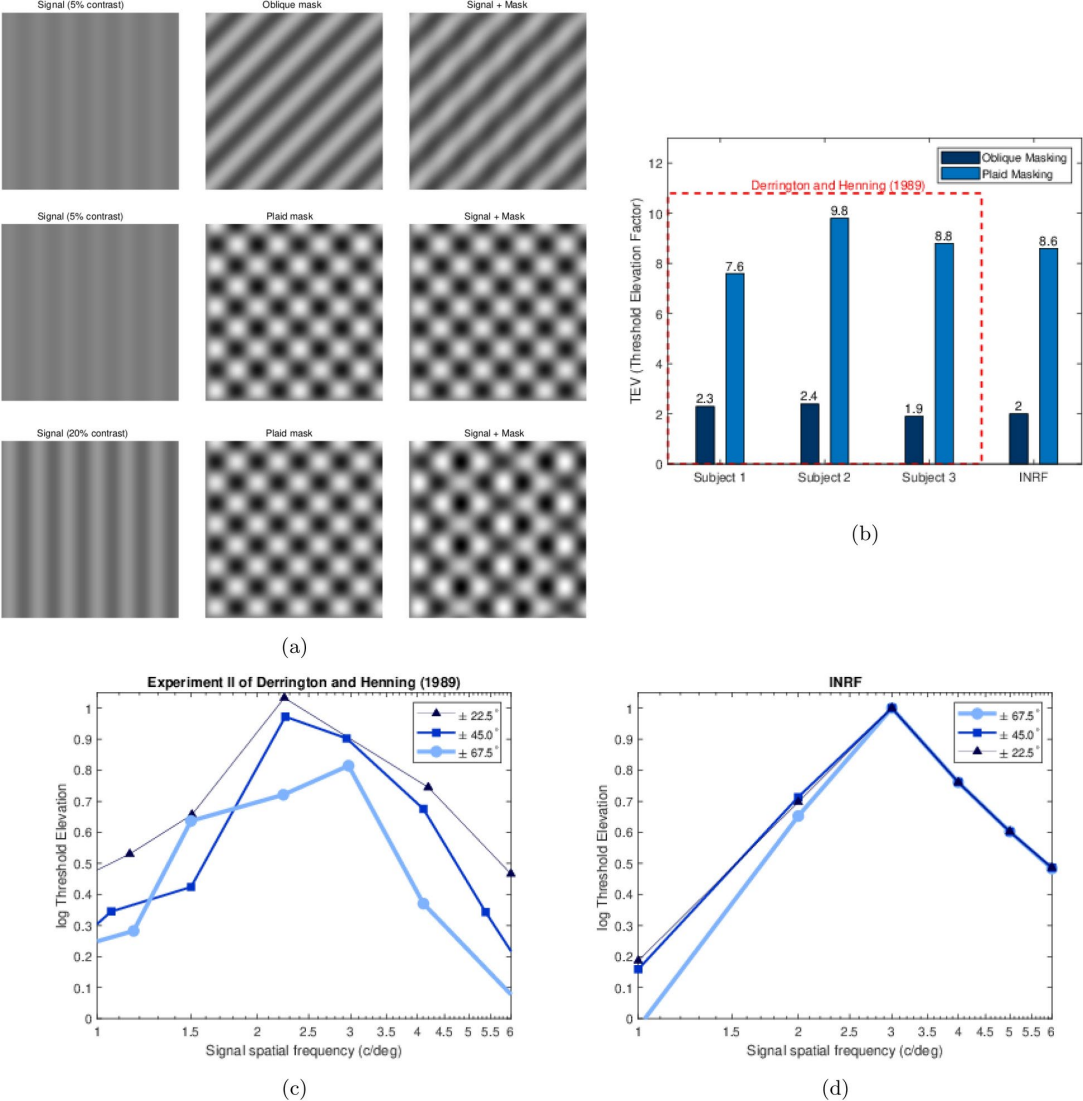
The INRF model reproduces the plaid masking data of Derrington and Henning^16^ . (**a**) The minimum signal contrast necessary to tell apart the image with signal and oblique mask (top right) from the signal with just the mask (top center), is not enough for the plaid mask case (middle row), for which the minimum signal contrast needs to be much higher (bottom row); this is the phenomenon that Derrington and Henning^16^ observed and which the standard model can’t explain. (**b**) Data from Experiment I in Ref.^16^ and INRF result, showing that the INRF model makes a prediction about the increase in threshold contrast from the oblique to the plaid masking case that is very close to the average of the results of Experiment I in Ref.^16^, and which the standard model can’t account for. (**c**) Data from Experiment II in Ref.^16^, showing how masking is a function of the spatial frequency of the mask, for plaid masks of three different orientations; the standard model can’t explain this data either, as it predicts that the $\pm 45^{\circ }$ maskers should produce much less masking than the $\pm 22.5^{\circ }$ maskers, and that the $\pm 67.5^{\circ }$ maskers should produce virtually no masking. (**d**) The INRF model, on the other hand, is able to predict qualitatively the data from Experiment II in Ref.^16^, shown in panel (**c**).

**Fig. 2:**
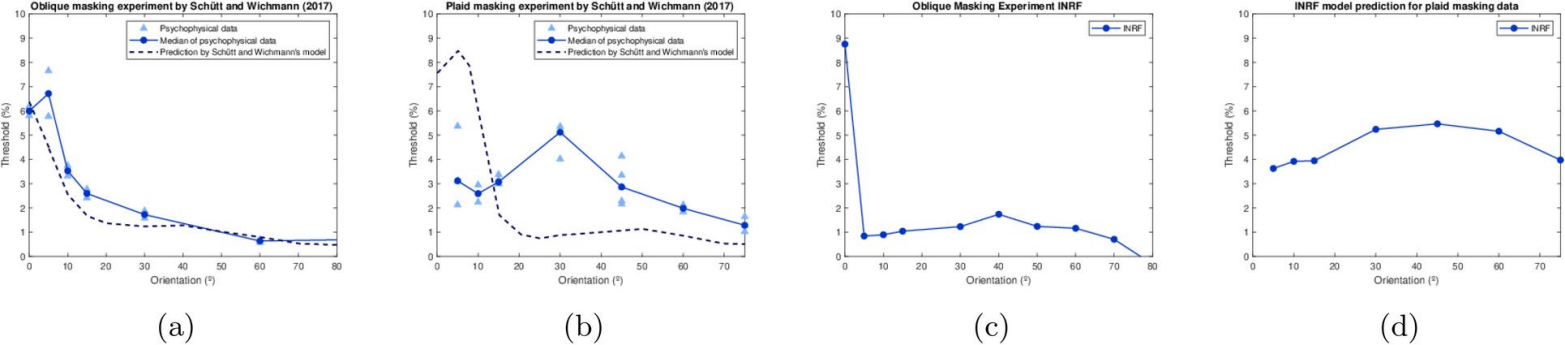
The INRF model reproduces the plaid masking data of Schütt and Wichmann^9^. (**a**) Oblique masking data and model prediction in Ref.^9^. (**b**) Plaid masking data and model prediction in Ref.^9^: here we can see that the standard model fails. (**c**) Oblique masking prediction by the INRF model, which reproduces qualitatively the experimental data shown in panel (**a**). (**d**) Plaid masking prediction by the INRF model, which reproduces qualitatively the phenomenon observed in the experimental data shown in panel (**b**), where the threshold remains large even as the orientation angle of the masking components departs substantially from $0^{\circ }$ (which is the orientation of the signal).

**Fig. 3:**
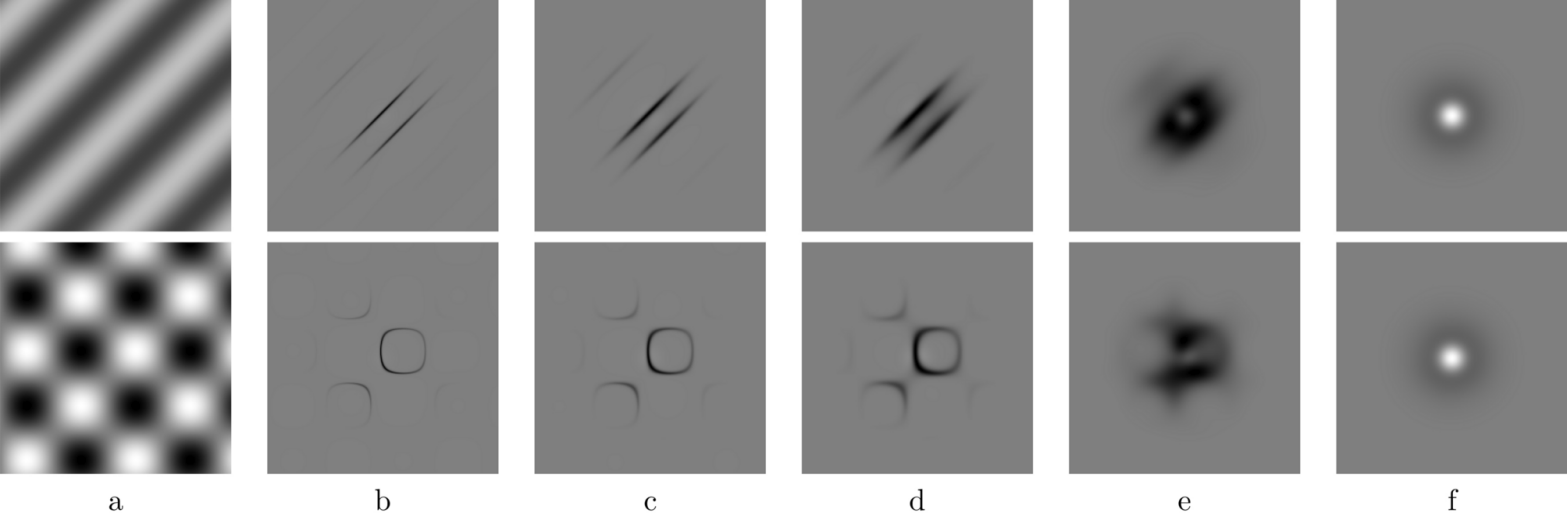
While the INRF model remains fixed, its associated receptive field (RF) changes with the input and substantially differs for oblique and plaid masking data. (**a**) Input visual stimulus. (**b**) Effective RF for our instance of the INRF model, computed for a neuron located at the center of the visual field; here the parameter *p*, that denotes the maximum slope of the sigmoid nonlinearity in the INRF model, has a value of 100. Notice how the RF is strongly adapted to the input, and therefore it’s quite different for the plaid than for the oblique mask. (**c**) RF when we reduce the slope of the sigmoid to $p=50$. (**d**) RF when we reduce the slope of the sigmoid to $p=25$. (**e**) RF when we reduce the slope of the sigmoid to $p=5$. (**f**) RF when we reduce the slope of the sigmoid to $p=1$, which means that we are in the linear case: notice how the RF has the shape of a Difference of Gaussians, and now it’s the same for both inputs.

We performed a brute force search parameter optimization in order to fit the average of the data in Experiment I in Ref.^16^, considering signals and masks of 3 cpd and a FOV of 3 degrees, and we obtained the following values: $\sigma _m=0.038$ deg, $\sigma _w=0.111$ deg, $\sigma _g=0.038$ deg, $\lambda =5$, $p=100$. These are the values used in all our tests with the INRF model, i.e. also to reproduce the data in Experiment II in Ref.^16^ (Fig. 1), to reproduce the oblique and plaid masking results of Schütt and Wichmann^9^ (Fig. 2), and to show how the RF associated to the INRF model changes substantially from an oblique to a plaid mask input (Fig. 3).

### Receptive field of an INRF unit

 In the examples in Fig. 3, the linear RF corresponding to the INRF model for a given input was computed in the standard way: as the weights to be applied to the input stimulus to compute the response^46^, which in nonlinear systems corresponds to a row of the Jacobian matrix^10^. From Eq. (1) for the *i*-th mechanism we have the following dependence in space (*j* index):

2 $$\begin{aligned} RF_{ij} = \frac{\partial O_i}{\partial I_j} = m_{ij} - \lambda w_{ij} f'(I_j - \sum _k g_{ik}I_k ) (1-g_{ij}) \end{aligned}$$

## Results and discussion

We begin with the data reported in Experiments I and II of Ref.^16^, where observers were presented with two images, one with signal plus mask and the other only with the mask, and they had to indicate which one contained the signal (Fig. 1a). In the following we apply a fixed instance of the INRF model to both images and then compute the RMSE between the responses; this value will be our proxy for the perceived visual difference between the two images. We choose to instantiate the INRF model with elements consistent with retinal physiology, using 2D Gaussians for the spatial connectivity kernels and a sigmoid for the nonlinearity, leaving us with five free parameters. We obtained a good fit to the data in Experiment I in Ref.^16^, see Fig. 1b —to our knowledge this is the first successful attempt at capturing the “super-additivity” of masking shown by a plaid pattern relative to its components in isolation.

To account for the data of Experiment II we use the same parameters that were optimised for Experiment I—no additional optimisation. The results in Fig. 1d again show that the INRF model makes predictions qualitatively similar to the plaid masking data reported in Ref.^16^ (Fig. 1c).

Next we take the exact same instance of the INRF model as above, again without changing any of its parameter values, and test it on the oblique and plaid masking data reported by Schütt and Wichmann^9^. The authors proposed a vision model based on the SM that is able to reproduce oblique masking but not plaid masking results (Fig. 2a,b), while the INRF model is consistent with both (Fig. 2c,d). Figure 3 shows that, while the INRF model parameters remain fixed, its associated receptive field (RF)—defined as the input perturbation that produces a maximal output^10,46,47^— changes with the input and is substantially different for oblique and plaid stimuli. In Fig. 3f we see the behaviour of the INRF with purely passive dendrites and it reduces to a standard difference-of-Gaussians RF *independent* of the input image. From (e) to (b) we move towards more and more active dendrites and stronger input *dependent* nonlinear RFs. This is consistent with the SM^9^ able to predict oblique and plaid masking *separately*, with very different parameters, but not concurrently with the same parameter set as INRF is able to do. It is important to note that a center-surround RF is not only obtained when de-activating the nonlinearities of the dendrites by modifying the slope of $f(\cdot )$ as in Fig. 3f. It also happens for zero contrast backgrounds: for uniform images the argument of $f'(\cdot )$ in Eq. (2) is zero for every location when using normalized kernels *g*, so $f'(0) = p/2$ and the equivalent RF reduces to a difference of Gaussians. The center-surround RF for zero-contrast images means that the model proposed here also reproduces the band-pass behavior at threshold level in the classical Contrast Sensitivity Function^2^ with the same set of parameters used for the plaid masking experiments.

To conclude we speculate about the anatomical locus of our INRF model instance and potential future models of early spatial vision.

First, our INRF model choices are consistent with retinal physiology—for reduced contrasts, using 2D Gaussians as connectivity patterns makes the INRF approximate a difference-of-Gaussians linear RF^39^: This invites the speculation that the behaviourally relevant nonlinearities responsible for the strong plaid masking may happen as early as in retinal ganglion cells (RGCs). Furthermore, it supports the INRF as a better model for dynamic retinal RFs^48^ given the deficits of models that begin with linear filtering^49^. In this sense, it will be interesting to compare the INRF formulation with the very recent work of^50^, where RGC output is predicted through a three-layer convolutional neural network (CNN) that was trained to fit neural responses to natural scenes, achieving an accuracy that is substantially higher than that of other successful approaches for the retinal neural code; however, this CNN model employs the ReLU function for rectification and is feed-forward, which means that it uses fixed nonlinearities and does not consider bAPs, so it may be the case that its outputs are not nonlinear enough to predict the plaid masking phenomena with sufficient accuracy.

Second, we are hopeful that our results offer a new avenue for developing more complex, two-stage models of spatial vision, incorporating the INRF as a pre-processing step to a traditional (potentially modified) SM. Such a future hybrid cascade model of spatial vision promises to account for the data the SM captures and in addition the data beyond the capabilities of the SM, such as plaid masking, captured by the INRF. Note that this suggestion of ours is unlike previous attempts trying to extend the SM with additional nonlinear post-processing subsequent to the SM: Instead we suggest it may be beneficial to augment the SM with a preceding stage of INRF form. In spirit, this is in line with Derrington and Henning’s idea expressed in 1989 that plaid masking may be the result of “distortion products” arising prior to the formation of the selective spatial-frequency and orientation channels in the SM. However, they only considered comparatively benign static non-linearities and concluded that those were unable to account for the observed plaid masking behaviour. Here, on the other hand, we show that an input-dependent rather than static non-linearity, the INRF, can account for the observed plaid masking behaviour.

## Data Availability

All data generated or analysed during this study are included in this published article.
